# The Legacy of Sport Events for Emerging Nations

**DOI:** 10.3389/fspor.2022.926334

**Published:** 2022-07-12

**Authors:** Brendon Knott, Cem Tinaz

**Affiliations:** ^1^Department of Sport Management, Cape Peninsula University of Technology, Cape Town, South Africa; ^2^School of Sports Sciences and Technology, Istanbul Bilgi University, Istanbul, Turkey

**Keywords:** sport events, legacy, emerging nations, mega-events, FIFA World Cup, Olympic Games

## Abstract

Large-scale and mega sport events (SMEs), such as Olympic Games and FIFA World Cups, have been more frequently hosted in emerging nations. Bidding and hosting SMEs is considered an object of policy for many emerging nations, with SMEs viewed as key factors in local and national development strategies. This has largely been driven by the assumption that their legacy provides solutions to economic, social, cultural, or political challenges. A variety of legacies have predominated the literature over the past two decades, however it is proposed that there is a difference in the types of legacies anticipated or realized within emerging nations. This exploratory study therefore aimed to determine the types of legacies anticipated or realized by emerging nations as a result of hosting sport events, and to determine if these differ from those of established nations. A systematic literature review followed the PRISMA approach to identify and select peer-reviewed articles that focused on legacies from major and mega-events hosted in emerging nations. A set of 97 publications were analyzed qualitatively to reveal the key legacy themes. The findings confirm legacy as a growing body of knowledge in emerging nations, aligned with increasing event hosting. The findings reveal insights on the extent of literature on this topic in emerging nations, including the major nations, events, authors and publications represented. While the paper cannot determine unique legacies for emerging nations, it identifies key legacy focus areas for these nations, primarily: social development; politics, soft-power and sport-for-peace; the economics of tourism, image and branding; infrastructure and urban development; and sport development. This paper proposes a conceptualization of key legacy areas for emerging nations and proposes future research themes. The paper is unique in its highlighting of the significance of legacy outcomes for emerging nations from the hosting of sport mega-events. It therefore contributes to a more nuanced understanding of and imperative for legacy from sport events globally.

## Introduction

The devolution of wealth and power from the major developed countries to the fast-developing countries in Africa, Asia, the Middle East, and South America has been one of the most significant outcomes of the twentieth century (Grix et al., 2019). The rapid expansion of most emerging economies is a distinguishing feature of these countries. Because they have modeled or been influenced by the many commercial sport successes of the established economies in North America and Europe, increased globalization has opened up new opportunities for sport leagues, teams, and manufacturers in emerging markets. This is especially true for sports leagues, teams, and manufacturers in emerging markets. In particular, the increased bidding for and hosting of large sporting events in emerging markets is a prominent manifestation of this. In a number of developing countries, signature or sport mega events (SME) have emerged as important components of local and national development agendas. Host cities are seeing increased tourism, local investment, and employment as a result of hosting these events, however the likelihood of truly lasting legacies is uncertain.

Emerging nations are those countries that are making investments in more productive infrastructure and human capital. They are moving away from their conventional economies, which have been based on agriculture and raw material exports. As a result, they are rapidly industrializing and transitioning to a free market or mixed economy (Morgan Stanley Capital International, 2021). The majority of emerging-market leaders aspire to improve the overall standard of living for their citizens in their countries (Tinaz and Knott, 2021). The Morgan Stanley Capital International Emerging Markets Index (Morgan Stanley Capital International, 2022) currently includes data from 24 different nations, Brazil, Chile, China, Colombia, Czech Republic, Egypt, Greece, Hungary, India, Indonesia, Korea, Kuwait, Malaysia, Mexico, Peru, Philippines, Poland, Qatar, Saudi Arabia, South Africa, Taiwan, Thailand, Turkey and the United Arab Emirates. These countries have similar indicators regarding sustainable economic growth, monetary policy and the maintenance of price stability, fiscal discipline, the state of the debt and trade, and the current accounts' balance.

The majority of these countries have confronted challenges that are vastly different from those faced by the established western states. Aside from the prevalent challenges of social and economic underdevelopment, several of these countries have recently witnessed political and ideological regime changes and worldwide isolation due to their political standing. Over the past several years, we have seen a shift in the sports sector, particularly in hosting sporting events, away from the rich western countries toward the developing world.

The globalization of the sports business has resulted in enhanced benefits and broader prospects for the industries of emerging nations (Tinaz and Knott, 2021). The use of sport as a vehicle to achieve social, economic, cultural, political, technological, and environmental objectives by decision-makers or investors is undeniably widespread throughout the world's emerging markets. The countries also acquire international recognition as a result of their sports-related efforts. Most emerging nations recognize sport's benefits for social and economic development. Attempts are being made by both the public and corporate sectors to harness the athletic potential in various forms to develop and promote their respective societies and stimulate their respective economies. As a result, politicians, event organizers, and other influential stakeholders seek legacy outcomes from SMEs (Brittain et al., 2017).

In the past two decades, interest in sport event legacies has grown exponentially. Thomson et al. (2019) noted that since 2012, there has been a considerable growth in the amount of literature on large-scale sport event legacy in the sports and event management fields. Yet, a relatively small number of systematic reviews or synthesizes of sport event legacy research have been published (Thomson et al., 2019).

Historically, the academic study of legacy has been predicated on the notion that it can be used to address economic and social problems as well as cultural, historical, and political challenges (Byers et al., 2020), which makes it particularly appealing to developing countries' development aspirations. In the literature, there are many distinct types of legacies that have predominated, including economic; social; cultural; environmental; health; sports participation; infrastructure; politics; tourism/destination branding; and security (Byers et al., 2020). It is difficult to assess the legacy of a person or organization due to the fact that it is a “complex, fluid, and contentious term that is likely to be realized differently” across a variety of situations based on socio-economic and political aspects (Brownill et al., 2013).

In several cases, there is evidence of a difference in legacy realization or objectives between developing and developed countries. Grix et al. (2019), for example, draw attention to the political legacy of SMEs, claiming that they have evolved into the ideal soft power project for emerging economies. According to Heslop et al. (2013), SME is “a fast-track to world recognition and reputation development” for rising countries, and this is supported by both the political and tourism/destination branding legacies (p. 13).

Consequently, the purpose of this research article is to provide answers to the following questions: What types of legacies do emerging nations anticipate to realize as a result of hosting SME? and Do the legacy expectations of emerging countries differ from those of established nations?

## Sport Events and Their Legacies

Sport events take place on a variety of levels or scales, with the “mega-event” the largest of these. Legacy has emerged as an important consideration in the development of SMEs (Spracklen, 2012), even if there has been a greater recognition of unintended repercussions (Cornelissen et al., 2011). Beyond the immediate benefits of sporting mega-events, many increasingly propose a longer-term focus on building legacies from such events (Cornelissen et al., 2011; Chappelet, 2012), with legacy being of either a planned or unplanned character (Cornelissen et al., 2011). Cornelissen et al. (2011) emphasized the necessity of understanding and assessing the legacies of sporting mega-events.

The legacy of major sport events has risen in relevance in recent years, garnering attention from both academics and practitioners alike (Preuss, 2019). The growing interest in examining the legacy of SMEs has to a large extent replaced the debate on mega-event impacts (Grix, 2012; Graeff et al., 2021). The notion of “legacy” is considered “multi-faceted and far-reaching” (Chappelet, 2012). Preuss (2007) devised a legacy cube with three dimensions: the past, the present, and the future. The paper pointed out that legacies can be deliberate or unexpected, as well as positive or negative, and that both are possible. It also distinguished “soft” legacies, such as incorporeal or psychic communal benefits, from “hard” legacies, such as infrastructure. This led to the formulation of the most widely accepted definition of sport event legacy as:

“… all planned and unplanned, positive and negative, concrete and intangible structures generated for and by a sporting event that last longer than the event itself, regardless of the time and space in which they were created.” (p. 211).

Although there is some agreement on the definition of legacy, what it entails, and how it should be conceptualized, there is still disagreement on how it should be measured (VanWynsberghe, 2016), with Preuss (2007) advocating the importance of future research attempting to develop more generic approaches and methodologies in order to address this.

It is because of this inability to measure legacy with any precision that some authors have urged a shift in emphasis to a more “systematic and purposeful” approach, referred to as “leveraging” (Grix, 2012). Leveraging refers to short-term operations carried out by event hosts, as well as long-term activities carried out before and after the event, in order to realize aims or planned legacies.

In the past decade, there has been increased criticism of the negative potential from hosting a mega-event. Critical questions and concerns have been expressed concerning the expanding expense, feasibility, long-term legacy, and repercussions of SMEs (Byers et al., 2021). Preuss (2019) explained that the costs of hosting and debate over a host government's expenditure of public funds, has made corruption a real possibility. This, together with corruption allegations linked to the FIFA and International Olympic Committee (IOC) hosting selections, has led to increased public and media scrutiny of the benefits of SMEs for the host. In response, the IOC established the Sustainability and Legacy Commission in 2015, responsible for consulting with, coordinating with, and monitoring the legacy of the Olympic Games (International Olympic Committee, 2017). Candidate cities are now required to track their legacy for several years after the Olympic Games as part of their host city contract (International Olympic Committee, 2017).

Although event impact studies have traditionally concentrated on visible or “hard” outcomes such as economic growth, infrastructure development, and tourism promotion, less tangible outcomes such as advantages to a country's image and identity are gradually being recognized. Similarly, there has been an increasing recognition of social legacies. Minnaert (2012) asserted that social legacies might occur at the personal level, such as health benefits and skill acquisition or at the community level, such as improved links and cooperation between community members, particularly from different backgrounds. Ma and Kaplanidou (2017) emphasized the time dimension of social legacies and explained how they could manifest themselves before, during, and after a particular event.

The literature reveals a wide variety of different types or categories of legacies that could result from sport events. Chappelet and Junod (2006) compiled these into five types or themes, as follows:

- Sporting legacy: e.g., sporting facilities and related infrastructure upgrades; and an increase in sport participation, support and sponsorship.
- Urban legacy: e.g., changes made to the urban structure of the host city as well as the development of new urban districts and specialized areas.
- Infrastructural legacy: e.g., networks, ranging from transport to telecommunications, which are renovated or developed for a mega-event; access routes by air, water, road or rail; and the modernization of basic services, such as water, electricity and waste treatment.
- Economic legacy: e.g., changes in the number of permanent jobs created and changes in the unemployment rate; economic investment opportunities; foreign investment attraction; and small business development/ entrepreneurship; the increase in tourists to a host region that stimulates the local economy.
- Social legacy: e.g., nation building and contribution to national pride; changed perceptions of residents; education; racial harmony; and environmental awareness.

Cornelissen et al. (2011) added three more legacies to this set, namely:

- Environmental legacy: e.g., reducing carbon footprint; integrating greening principles; and climate-responsiveness.
- Political legacy: e.g., the promotion of democracy, human rights and improved governance; enhancement of capacity within the public sector; improvements in skills and human resources capital in public and private sectors; interventions by government or non-government organizations.
- Image/ branding legacy: e.g., destination-profiling; host-region exposure; setting or changing the image of a host destination; changes in tourist image and reputation; and brand marketing for a host region.

**Figure 1:**
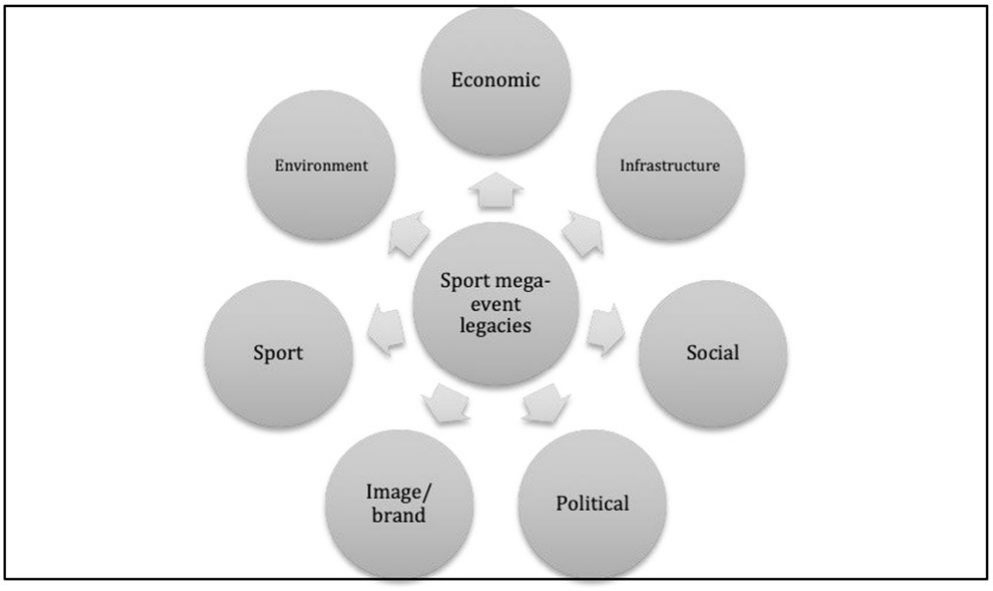
Sport mega-event legacies (adapted by authors, from Cornelissen et al., 2011, p. 311).

Figure 1 illustrates these different aspects of legacy. Adapted from Cornelissen et al. (2011), it uses the five aspects of Chappelet and Junod (Chappelet and Junod, 2006), but combines urban legacy with infrastructure, and adds the additional three elements discussed above.

According to Preuss (2015), the five most frequently mentioned legacy areas are economics (including infrastructure), social, sport, and culture. Grix et al. (2017) added to this list: urban regeneration, national pride/ feel-good factor, increased involvement and participation in physical activity, international reputation and 'soft power'. Increasingly, attention is being paid to the possibility that sporting events and their legacies could serve as a platform to address global concerns and effect social change (Byers et al., 2021). For example, the United Nations (UN) has highlighted the significant role that sport plays in promoting the UN 2030 Agenda for Sustainable Development.

Preuss (2007) noted the following three issues that create challenges in developing a standardized legacy measurement approach:

- The same event creates different legacies if staged twice in one city/ nation: Both the events and the cities/ nations staging them are continuously developing such that the event has different requirements at a later stage of hosting and the host city/ nation has different environmental factors to consider (e.g., FIFA Football World Cup in Germany hosted in 1974 in comparison to 2006).
- Different events create different legacies if staged in the same city/ nation: Differing infrastructural requirements, social interests, media exposure, and location requirements result in a unique legacy. For example, Rio de Janeiro hosted both a FIFA World Cup in 2014 and an Olympic Games in 2016. Yet, the legacies attributed to these events differ substantially.
- The same event creates different legacies in different cities/ nations: This may be a result of a number of factors, including different infrastructure of the cities/ nations and the political targets pursued for the event. For example, a FIFA Football World Cup held in Germany in 2006 may yield very different legacies compared to the same event held in South Africa in 2010.

These challenges led the writers to propose that perhaps emerging nations may produce legacies or at least aim to produce legacies more akin to each other than from more developed nations.

## Materials and Methods

This research aimed to draw attention to the subtle nuances and distinct variations in the sport event legacy discourse among emerging nations by obtaining research findings from peer-reviewed, academic journal-based literature. As a means of accomplishing this, the authors conducted a systematic qualitative review of scholarly articles that empirically investigate the legacies of sporting events hosted by emerging nations and that have been published within the last 20 years (between 2002 and 2022). Literature reviews, particularly for emerging topics, are becoming increasingly accepted as worthwhile research endeavors in the social sciences field (Pickering and Byrne, 2014). Although there has been some research into event legacy literature in emerging nations, there has been no systematic review of this literature to date. According to Thomson et al. (2020), researchers in event legacy studies are disproportionately concentrated in Western countries, and legacy research has been disproportionately biased in terms of geography.

This study was structured following the PRISMA (Preferred Reporting Items for Systematic Reviews and Meta-Analyses) guidelines for conducting systematic reviews and meta-analyses (Liberati et al., 2009). An official protocol for the electronic search was devised, which was limited to sources in the English language. The research topic guided the selection of databases, as did the likelihood of those databases to hold articles of relevance to the study. Articles were searched for using the following databases: SCOPUS, SPORTDiscus, Web of Science, Google Scholar, Business Source Complete (EBSCO), Science Direct (Elsevier), and Emerald. We used the phrases “sport event” and “legacy” to search for full-text, peer-reviewed academic journal papers published between 2000 and 2022. The starting date of 2000 was chosen as it symbolizes the period when conceptual development and debate surrounding sport event legacy intensified (Thomson et al., 2019). The computerized search produced 270 sources; once duplicates throughout the database were removed, a manual screening of these sources was conducted to select only the papers that referred to emerging nations. The final number of sources selected was 96.

The sources were captured in an Excel spreadsheet, with the following bibliographic details captured from each source: title; authors; 1^st^ author nationality; journal; year; event focus; country focus; and keywords. A quantitative analysis of the bibliographic data was conducted using descriptive statistics (i.e., frequencies), with matrices, tables or graphs produced to reflect the key findings.

A manual, qualitative assessment was conducted in order to determine the legacy focus of each article. The seven legacy types proposed by Cornelissen et al. (2011) were used as the legacy categories. The authors assigned the legacy focus, and in some cases a secondary legacy focus, after reviewing the title, keywords and abstract of each source. This review process also helped to eliminate any papers that did not fit the ambit of this investigation, such as articles focused only on theoretical constructs of legacy, rather than relating to an event or emerging nation context.

Once the legacy focus was assigned, a further, deeper analysis of the sources was conducted, combining the bibliographic findings, to allow for the discovery of key legacy themes from the sources. The findings are detailed in the following section, with the deeper analysis forming the basis of the discussion that follows.

## Findings

The section above indicated that a final sample of 97 peer-reviewed articles on sport event legacy in emerging nations, published between 2000 and 2022, were included in the systematic literature review. This section sets out the findings from the quantitative and qualitative analysis of these articles in terms of: (1) bibliographic details; and (2) types of legacy.

### Bibliographic Details

#### Lead Researchers and Location of Universities

The nationalities of the first authors, according to their university affiliation, represented 22 different nations. Only 13 of these are emerging nations. The nations with the largest representation were: South Africa (24), UK (17) and Brazil (14). These three nations alone accounted for 57% of the articles. The first authors with the most papers were: Knott, B. (5 papers); Lee (2019) (4); Cornelissen, S. (3); Kim et al. (2006) (3); and Rocha, C. (3).

#### Year of Publication and Journal

**Figure 2:**
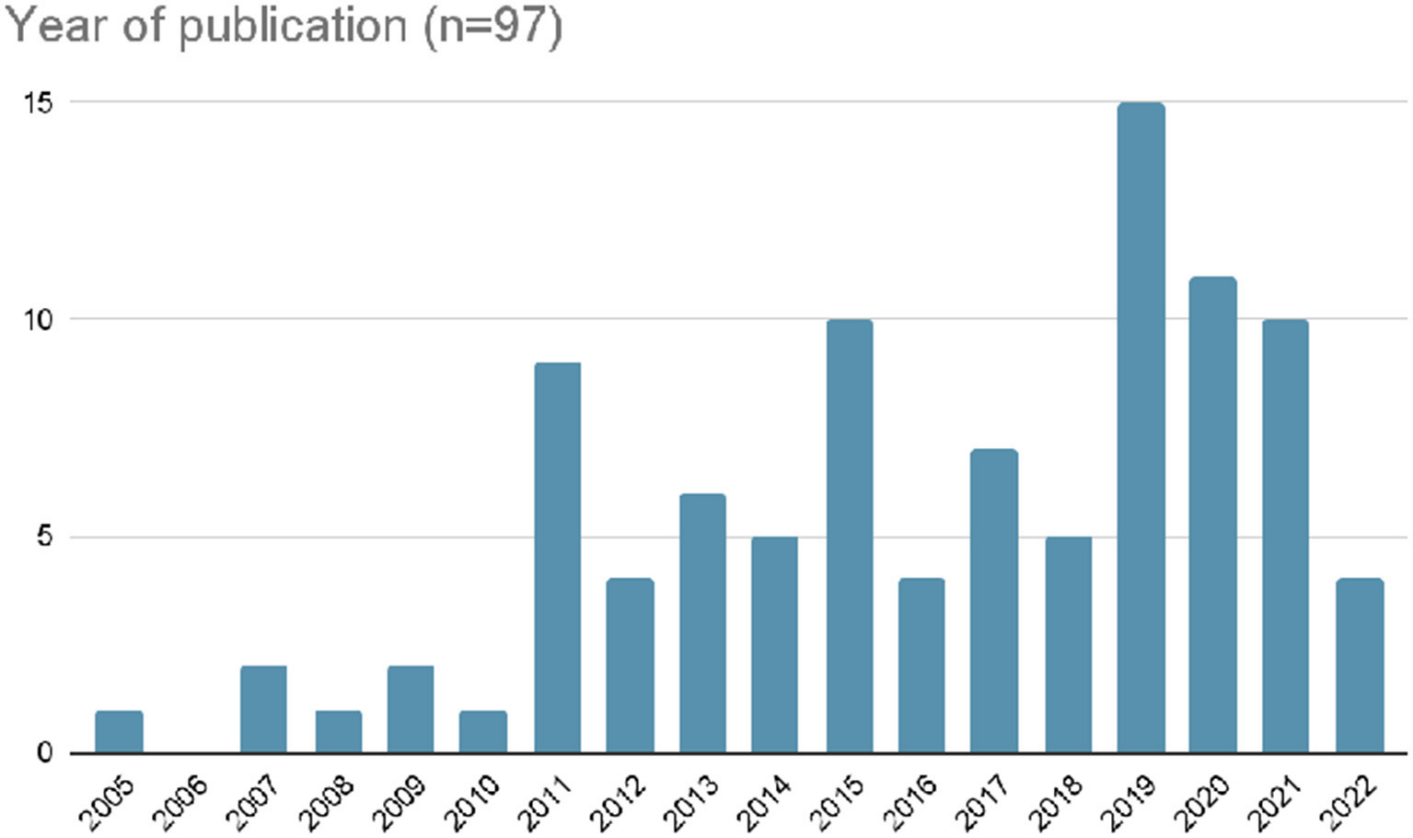
Year of publication.

The Journals with the most articles were: Sustainability (6); Leisure Studies (5); and Development Southern Africa (4). The years of publication with the most articles were: 2019 15); 2020 (11); 2021 (10) and 2015 (10) [see Figure 2]. Thirty-eight percent of articles were published between 2019 and 2021. Only 7% of articles were published before 2011.

Table 1 also clearly shows the impact of the 2010 FIFA World Cup (South Africa), the 2014 FIFA World Cup (Brazil); and subsequent mega-sport events in the following years that were held in emerging nations, such as the 2016 Olympic Games (Brazil), 2018 Olympic Games (South Korea), and 2018 and 2022 FIFA World Cups (Russia and Qatar respectively).

**Table 1:** Event type.

	**(*n* = 97)**
FIFA World Cup	44
Olympic and paralympic games	25
Sport mega event (multiple)	10
UEFA EURO	5
Major event (multiple)	4
Commonwealth games	2
University olympiade	2
Asian games	1
European youth olympic games	1
Formula one	1
Pan-American games	1
Tour de Taiwan	1

#### Event Type and Country Focus

Sport mega-events dominated the focus of the papers, with FIFA World Cups (44) and Olympic Games (25) combining to account for 71% of the events featured. A further 10 articles covered more than one sport mega-event in the article. The remaining mega and major events that featured are listed in Table 1.

A total of 12 emerging nations were the focus of the papers reviewed. The countries most focused on were: South Africa (29), Brazil (26), South Korea (8), Poland (5) and Qatar (5). South Africa and Brazil clearly dominated the article count, accounting for 57% of the papers. A further nine articles featured a combination of emerging nations. The full list of nations featured is found in Table 2.

**Table 2:** Country focus.

	**(*n* = 97)**
South Africa	29
Brazil	26
Various	9
South Korea	8
Poland	5
Qatar	5
China	3
Russia	3
Taiwan	3
India	2
Turkey	2
Colombia	1
Greece	1

### Types of Legacy

#### Keywords

**Figure 3:**
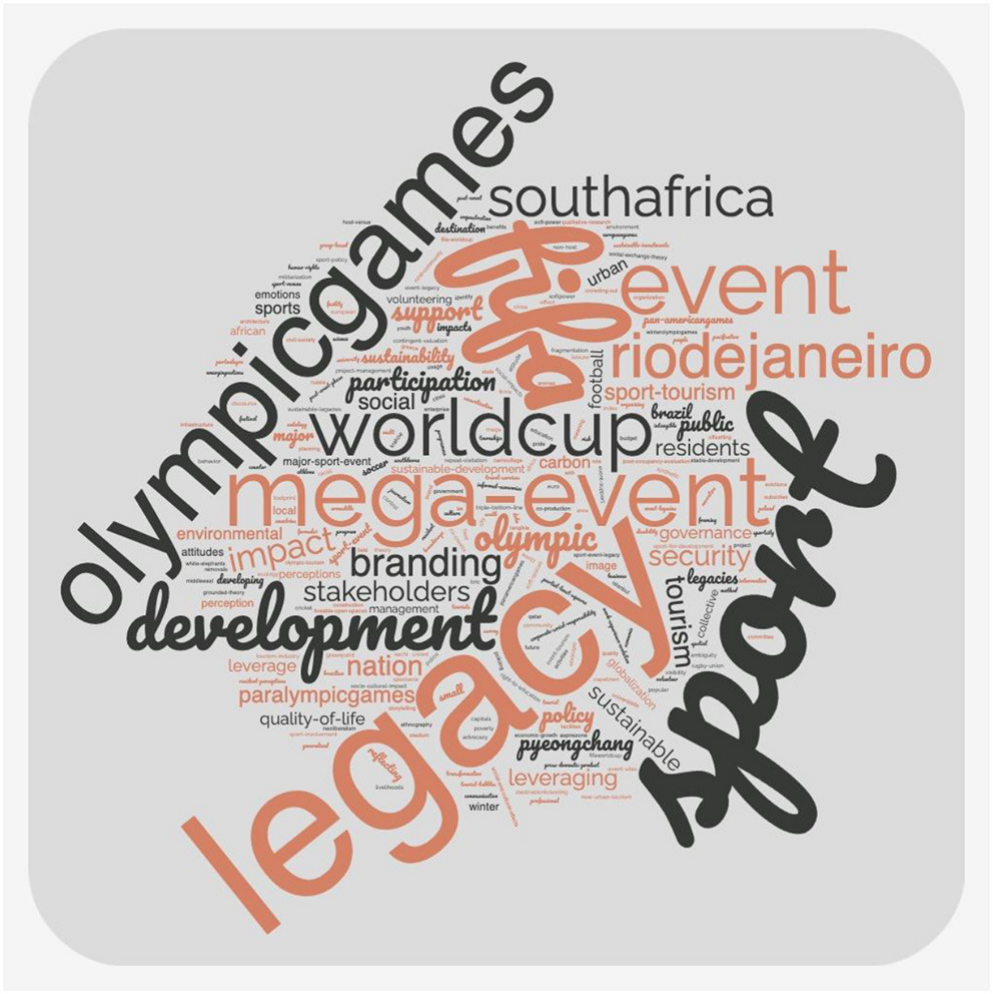
Keywords.

An analysis of the keywords listed in each of the papers revealed the following most frequently listed keywords not surprisingly: sport mega event/ mega event (85), legacy (55), World Cup/ FIFA World Cup (53), Olympic Games (20). Among the keywords that indicated a legacy focus, the following featured most commonly: sport participation (5), sustainable development (5), stakeholders (5), nation branding (4), sport tourism (4), quality of life (3) and social impact (3). Figure 3 displays a word cloud of the keywords. However, these represent very low numbers and indicate that most of the papers did not clearly specify a legacy focus within the keywords of the paper.

#### Legacy Focus

The writers assessed the legacy focus of each paper by reviewing the article title and its keywords, followed by the article abstract. If the legacy focus was still not clear, the full paper was then consulted. The authors used the classification of legacy types as compiled by Cornelissen et al. (2011), namely: economic; environment; image/ brand; infrastructure; political; social; and sport. Table 3 indicates the allocation of papers to the legacy types. Seven of the papers had a distinctive secondary legacy focus, so they were included in two categories.

**Table 3:** Legacy types.

Economic (incl. Tourism)	Antonio et al., 2011 Bondarik et al., 2020 Coakley and Souza, 2013 Duignan et al., 2022 Fourie et al., 2011 Gezici and Er, 2014 Kobierecki and Pierzgalski, 2022 Lee et al., 2013 Moyo et al., 2020 Rogerson, 2009 Tichaawa and Bob, 2015 Ziakas and Boukas, 2012
Environment (incl. sustainability)	Ermolaeva and Lind, 2021 Gulak-Lipka and Jagielski, 2020 Kim and Grix, 2021 Kim et al., 2006 Lee, 2019 Melo et al., 2014 Spanos et al., 2022 Talavera et al., 2019 Yoon and Wilson, 2019
Image/ brand	Allen et al., 2013 Hemmonsbey and Tichaawa, 2018 Knott et al., 2013 Knott and Hemmonsbey, 2015 Knott et al., 2015a Knott et al., 2016 Lee et al., 2005 Maiello and Pasquinelli, 2015 Swart et al., 2019
Infrastructure	Azzali, 2016 Azzali, 2019 Bason et al., 2015 Dendura, 2019 Gezici and Er, 2014 Kirby and Crabb, 2019 Lee, 2021 Lu and Lin, 2020 Malhado et al., 2013 Molloy and Chetty, 2015 Domareski Ruiz et al., 2019 Zawadzki, 2016
Political (Including security)	Black, 2007 Byun and Leopkey, 2020 Cornelissen, 2007 Cornelissen, 2011 Cornelissen, 2012 Curi et al., 2011 Dowse and Fletcher, 2018 Eisenhauer et al., 2014 Filho et al., 2018 Giulianotti and Klauser, 2010 Kobierecki and Pierzgalski, 2022 Majumdar, 2011 Ntloko and Swart, 2016 Pauschinger, 2020 Samatas, 2011 Sengupta, 2017 Włoch, 2020
Social	Al-Emadi et al., 2017 Al-Emadi et al., 2022 Azzali, 2016 Bob and Majola, 2011
	Bob and Swart, 2009 De Lisio et al., 2019 Graeff et al., 2020, 2021 Gursoy et al., 2017 Jaskulowski and Surmiak, 2016 Knott et al., 2015b Kolmakov, 2021 Kossakowski, 2019 Koutrou and Berber, 2021 Lee et al., 2013 Liu, 2016 Ma and Kaplanidou, 2017a, Ma and Kaplanidou, 2017b Maharaj, 2015 Ntloko and Swart, 2016 Patreze et al., 2020 Pillay and Bass, 2008 Prouse, 2012 Rocha et al., 2017 Rocha, 2020 Sullivan, 2018 Talbot, 2021 Tichaawa and Bob, 2015 Vico et al., 2019 Waardenburg et al., 2015 Xing and Chalip, 2012 Zawadzki, 2016
Sport	Bek et al., 2019 Feng and Hong, 2013 Kim and Kaplanidou, 2019 dos Santos, 2019 Reis et al., 2014 Reis et al., 2021 Ribeiro et al., 2021 Rocha et al., 2021 Sousa-Mast et al., 2013 Swart et al., 2011 Wodniak, 2021

**Figure 4:**
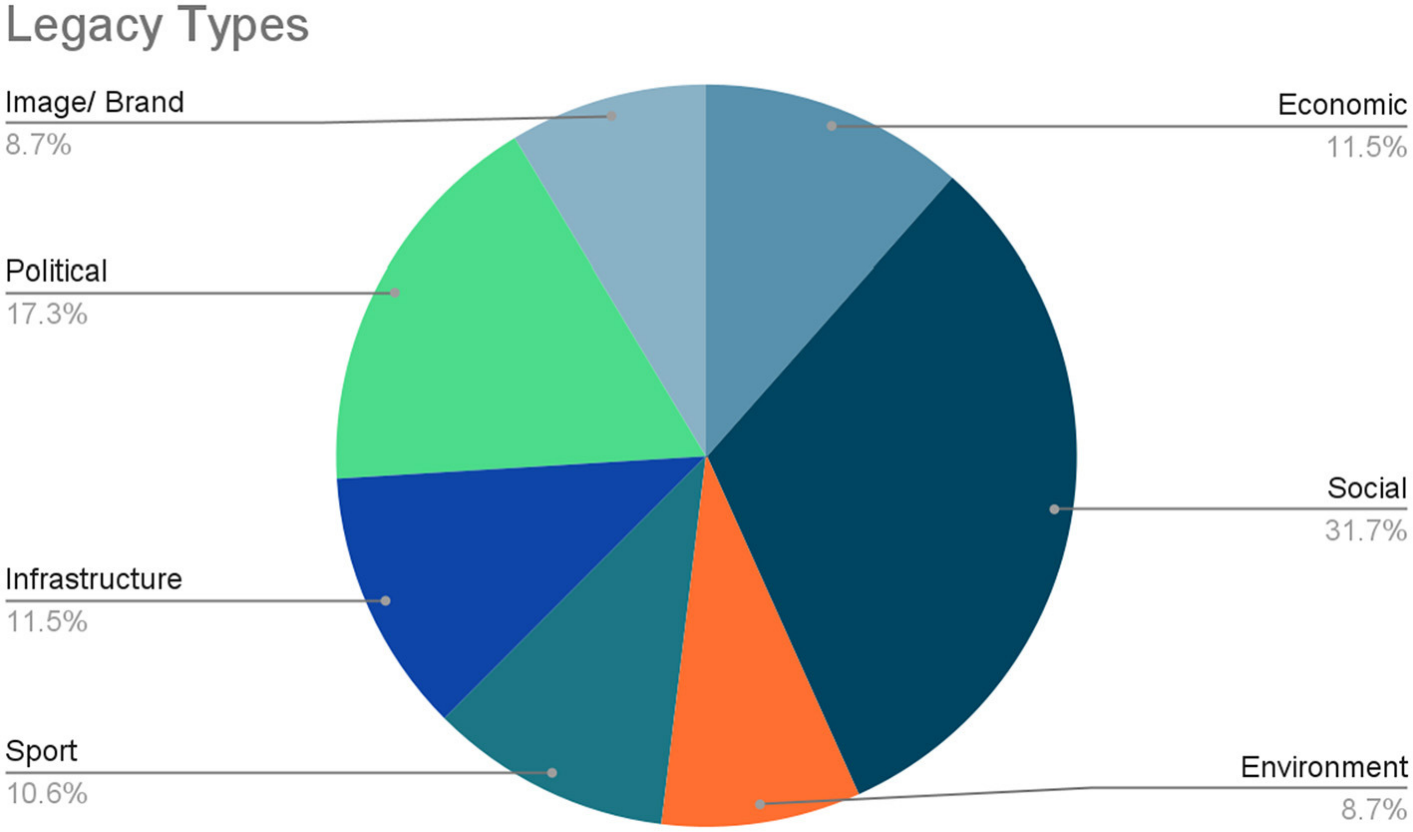
Legacy types.

As depicted in Figure 4, the papers were distributed as follows, from highest to lowest: social (31.7%); political (17.3%); economic (11.5%); infrastructure (11.5%); sport (10.6%); environment (8.7%); and image/ brand (8.7%).

These findings give an overall indication of the distribution of legacies from the papers reviewed. This distribution in itself does not reveal a unique legacy focus for emerging nations compared to the general legacy literature. However, the authors noted a few nuances within this distribution that may highlight key legacy focus areas for emerging nations. The following section discusses these nuances from the findings, providing deeper insights and meaning.

## Discussion

Although there are many similarities among emerging nations in terms of their socio-economic developmental status and challenges that they face, they also each face their own unique circumstances, priorities, policies and development agendas. Therefore, MSE legacy in these emerging nations must be understood within the context of each nation's social and economic sphere, as well as its historic and political legacy. This section now integrates a discussion of the exploratory findings with selected evidence and examples from the papers reviewed. The five key legacy focus areas form the structure of this discussion.

### Social Development

While social legacy was clearly the major legacy type featured, the category was by no means uniform in its legacy focus. In its broadest uniformity, the papers largely related legacies for local residents and populations most impacted by an event. There was an indication that within this legacy type, a focus on wellbeing or quality of life of residents (e.g., Ma and Kaplanidou, 2017), which is not specifically related to sport participation, may become a growing legacy focus in future.

Within this categorization, there were multiple examples of negative legacies, with examples of negative social ills attributed to event hosting (e.g., human trafficking) or disruptions to communities through resettlement programmes (e.g., from Brazil's 2014 FIFA World Cup). However, these may not be unique to emerging nations only. The issue of human rights as a legacy outcome was mentioned in the context of social legacies, but only in a few papers (e.g., Graeff et al., 2021). This is perhaps surprising as a number of emerging nations have faced global media criticism surrounding human rights issues highlighted through the hosting of a sport event.

What does appear to be a more unique focus within this legacy type for emerging nations, is a focus on social development. Whether emerging from a colonial past (e.g., Brazil), a repressive regime (e.g., Poland), isolation and fragmentation (e.g., South Korea) or legislated racial and societal divisions (e.g., South Africa), MSE have been embraced for their social unifying effect for many historically or currently divided populations. This is sometimes referred to as “nation-building.” Even with a focus on the future of MSE hosting, the legacy focus for Qatar's 2022 FIFA World Cup appears to be positive socio-cultural development initiatives (e.g., Al-Emadi et al., 2022).

### Politics, Soft-Power and Sport-for-Peace

A political legacy emerged as the second most common legacy type from the papers reviewed. It appears that emerging nations consistently expect mega-events to deliver on politically motivated aims. However, these aims can be divergent in their nature, from peace-related initiatives to global prestige and soft-power. For example, joint athlete participation in sport mega-events across the Korean peninsula has proved to be one of the sole means of bridging the divisions between the north and south, even normalizing relations to some extent. The international media narrative highlighted a unification story surrounding the Pyeongchang 2018 winter Olympic Games.

A different example, more akin to global prestige, is linked to Qatar and the 2022 FIFA World Cup. An international communication strategy was employed by Qatar to emphasize the host nation's role in contributing to international aid, conflict resolution, and peacebuilding in the region (Al-Emadi et al., 2022). This may also have been an aim to counter the largely negative publicity surrounding the lead up to the event, relating to its bidding process and the rights of migrant workers involved in the mega-event construction projects.

Although the South African example of historic division is rather different and based on racial classification and economic inequality, SMEs such as the 1995 Rugby World Cup and the 2010 FIFA World Cup, left a legacy as both socially and politically unifying catalysts, even if more symbolic in their effect (Black, 2007). The majority of the papers published in the immediate aftermath of the 2010 FIFA World Cup reflected on the social, historical and political context of the event, emphasizing the legacy of the event in national identity formation and political symbolism for the host nation. The government's social transformation aim was also highlighted as a legacy priority in papers that focused on South Africa.

As mentioned previously, it is not always easy to isolate the legacy types. An example of a political legacy that impacted economic, social and sport legacies, is that of Brazil. In Brazil, there was an intentional political strategy behind its government bidding for and hosting serial sport mega-events for both political and economic benefits. The hosting of sport mega-events impacted public policies, funding, and communities in host cities. While this may have been beneficial to a few sport sectors, it negatively disadvantaged certain population groups. It had adverse outcomes for Brazil's more excluded communities, while temporary funding was mainly channeled toward elite sport (Graeff et al., 2020).

In some instances, sport events are accused of being politically motivated from the perspective of the sport federation. Particularly in the case of SMEs, these global events could be seen as a means to promote globalization and a neoliberal legacy. Governance and the politics of development are particular issues affecting emerging nations. These aspects raise awareness of a more sinister side to the political legacies within emerging nations, mentioned particularly in the cases of Brazil's 2014 FIFA World Cup and Russia's 2014 winter Olympic Games.

A related theme under political legacy, according to the legacy model used, is security. Five papers reviewed were focused solely on investigating improved security, crime reduction, or security risk mitigations as a legacy. These were mostly focused on the events from Brazil and South Africa, but also various events among emerging nations. Also linked to political security, there was mention of negatively perceived legacies such as the pacification and militarisation of host populations (Prouse, 2012).

### The Economics of Tourism, Image and Branding

It is difficult to isolate different aspects of the economic legacy from sport events. While the model used in this study includes the tourism legacy as part of the economic legacy, others have preferred to separate these aspects. Furthermore, Byers et al. (2020) combined included destination branding as part of the tourism legacy. While the aim of this paper was not to define legacy types, it serves to highlight the connected nature of legacies.

Almost half of the economic legacy papers related specifically to a tourism legacy. Economic legacies reviewed were typically related to: economic growth; GDP increase; and small enterprise development. However, tourism-related economic legacies included: increased tourism budgets; new source markets; increased urban tourism; sport tourism development; improvements of travel services; and repeat visitation.

Although related as a distinct legacy type in the model used in this study, “image/ branding” (accounting for over 8% of publications reviewed) is closely related to tourism legacy. The case of South Africa's hosting of the 2010 FIFA World Cup is preeminent in its focus on this legacy, with seven (out of nine) papers focusing on nation branding as a legacy for South Africa from the 2010 mega-event. These papers indicated that the SME left a legacy of global branding gains for the host nation, providing a boost to its emerging status and aiding the development of its sport tourism industry (e.g., Knott et al., 2017).

Brazi, Poland and South Korea were also featured examples of image/ branding legacies for the host nation. For Poland, the hosting of the UEFA EURO 2012 was perceived as successfully showcasing the country's “new face” internationally as it emerged from its communist legacy. The event is believed to have strengthened the Polish image among visitors and football fans and enhanced its international competitiveness (e.g., Włoch, 2020). Similarly, through its hosting of events such as the 2002 FIFA World Cup (and subsequently the 2018 winter Olympic Games), South Korea aimed to portray its “global” identity - highlighting its economic liberalization and global prestige (e.g., Lee et al., 2005).

### Infrastructure and Urban Development

Infrastructure legacy emerged as another contested legacy within the emerging nation context. A combination of positive and negative legacies were explored from a range of examples, including Brazil, Colombia, Poland, Qatar, South Africa, South Korea, Taiwan and Turkey. A broad set of themes are explored within this legacy, such as: urban planning; event planning; local development; sustainable development; post-event occupancy/ usage; mega-project construction; architecture; mobility; liveable open spaces; and public facilities.

A key area for papers with a focus on infrastructure legacy was “sustainable development” (e.g., Gulak-Lipka and Jagielski, 2020). While large-scale infrastructure development has been a hallmark legacy for most sport mega-events globally, within emerging nations, these events have been more catalytic in nature. It appears that the sport events can play a central or focul role for broader development within the host nation. For example, UEFA EURO 2012 became a central point for many development projects in Poland, primarily relating to sport infrastructure development. However, in many instances, the infrastructure legacies reported were far beyond merely the sport infrastructure required to host the events. Public transport, urban development, housing and public facilities (including parks and recreational spaces) were all cited as examples.

There was a strong link between infrastructure legacy and environmental legacy, through the lense of sustainable development. While environmental legacy was the least of the legacy types featured in the analysis, the papers on this topic emphasized a legacy through sustainable events. They also highlighted the positive role the events can play in environmental communication and messaging.

### Sport Development

The key standout focus of a sport legacy was the focus on sport participation, which accounted for nearly half the papers. This does not appear to be unique to emerging nations, yet it is still a key legacy feature. Other legacy aspects related to: sport development; sport facility usage; sport involvement; corporate social responsibility; and support for future sport events.

However, it should be noted that it proved difficult to isolate the sports legacy. For example, some papers referred to sport-for-peace initiatives, although the focus was clearly aligned to political legacy. Furthermore, outcomes from these initiatives emphasized positive social legacies.

## Conclusion

This paper set out to ascertain if there is a difference in the legacy focus within emerging nations, by reviewing all peer reviewed journal articles on this topic that focused on examples from emerging nations. While not proposing that these nations be considered as a singular entity, this paper has highlighted the similarities across the papers reviewed and has drawn attention to the most pertinent examples.

The findings certainly highlights the need for a more critical assessment of sport event legacies in emerging nations. While the paper has attempted to isolate legacy types according to accepted frameworks, the findings indicate that legacies can very seldom be separated from each other. Legacies are certainly inter-connected. While of some merit for identifying differences from established nations, the broader legacy types reviewed in this paper are perhaps too broad in order to reflect the key legacy issues of importance for emerging nations. A deeper, qualitative analysis of the papers revealed nuances in legacy aims and delivery that highlights the differences within emerging nations more clearly.

**Figure 5:**
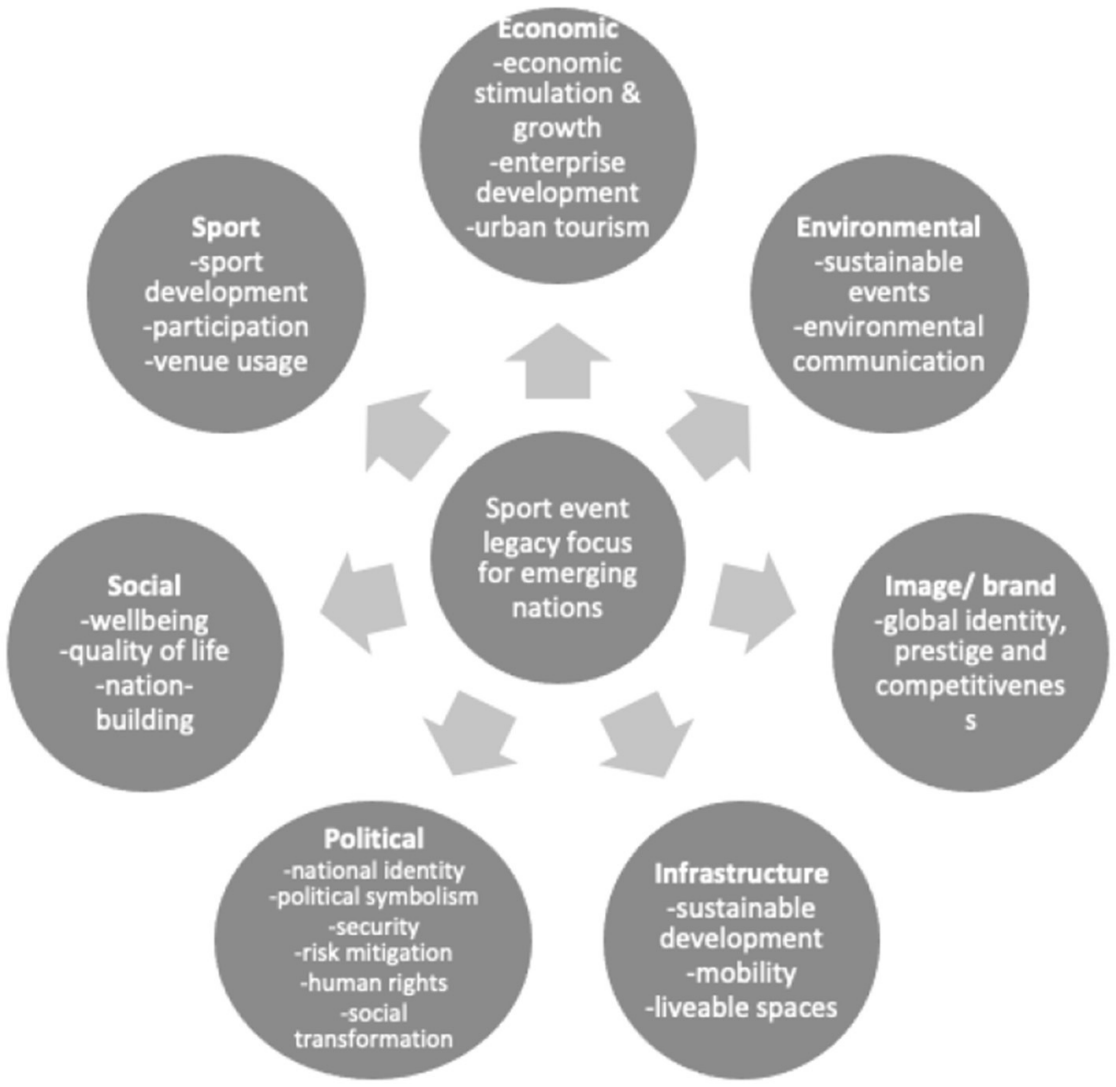
Legacy focus for emerging nations.

For example, this paper has revealed legacy focus areas that may be already or become the focus of event planning or legacy research in these nations. The list below (and depicted in Figure 5) indicates the authors' summary of the key legacy focus areas for emerging nations at present and into the near future, namely:

- Economic legacy: economic stimulation and growth; enterprise development; and urban tourism.
- Environmental legacy: sustainable events; environmental communication.
- Image/ brand legacy: global identity, prestige and competitiveness.
- Infrastructure legacy: sustainable development; mobility; and liveable spaces.
- Political legacy: national identity formation; political symbolism; security; risk mitigation; human rights; and social transformation.
- Social legacy: wellbeing; quality of life; and nation-building.
- Sport legacy: sport development; participation; and venue usage.

The authors recommend that future legacy papers consider the paradoxes of development within underdevelopment among the emerging nations. For example, there is often little critique of apparent positive legacies such as investments in world-class sport facilities that cause exclusion and the redirection of investment from other means of development. The opportunity costs associated with these legacies certainly need to be considered. Another broader critique of the legacy studies in emerging nations is the consideration of the host population's approval of the event. Many of the emerging nations are countries where democracy is not entrenched or where citizens have less say in the selection of events and the decisions surrounding the legacy aims. Greater citizen partnership and inclusion is therefore encouraged in the setting of legacy agendas. This paper has therefore laid the groundwork for future publications that follow this exploratory review, that aim to connect and examine the social fabric and underpinnings of these findings.

## Data Availability Statement

The original contributions presented in the study are included in the article/supplementary material, further inquiries can be directed to the corresponding author.

## Author Contributions

Both authors listed have made a substantial, direct, and intellectual contribution to the work and approved it for publication.

## Conflict of Interest

The authors declare that the research was conducted in the absence of any commercial or financial relationships that could be construed as a potential conflict of interest.

## Publisher's Note

All claims expressed in this article are solely those of the authors and do not necessarily represent those of their affiliated organizations, or those of the publisher, the editors and the reviewers. Any product that may be evaluated in this article, or claim that may be made by its manufacturer, is not guaranteed or endorsed by the publisher.
